# Supplementary material for: GATA1-deficient human pluripotent stem cells generate neutrophils with improved antifungal immunity that is mediated by the integrin CD18
Source: bioRxiv. 2024 Oct 11:2024.10.11.617742. Preprint. [Version 1] doi: 10.1101/2024.10.11.617742 (PMC11482877; doi:10.1101/2024.10.11.617742)
Supplement: Supplement 1 [file NIHPP2024.10.11.617742v1-supplement-1.pdf]

bioRxiv preprint doi: https://doi.org/10.1101/2024.10.11.617742; this version posted October 11, 2024. The copyright holder for this preprint (which was not certified by peer review) is the author/funder, who has granted bioRxiv a license to display the preprint in perpetuity. It is made available under aCC-BY 4.0 International license.

S1 Figure

A.

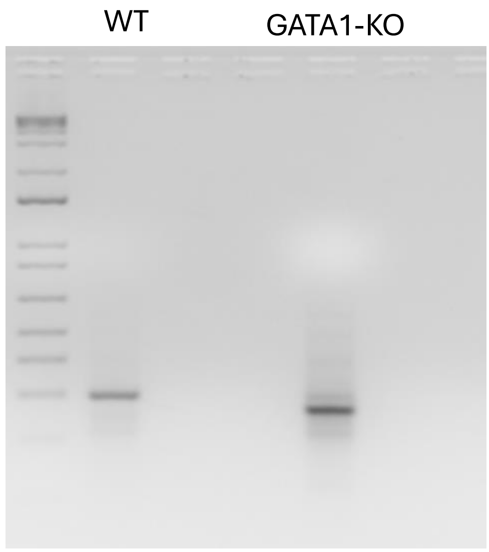

B.

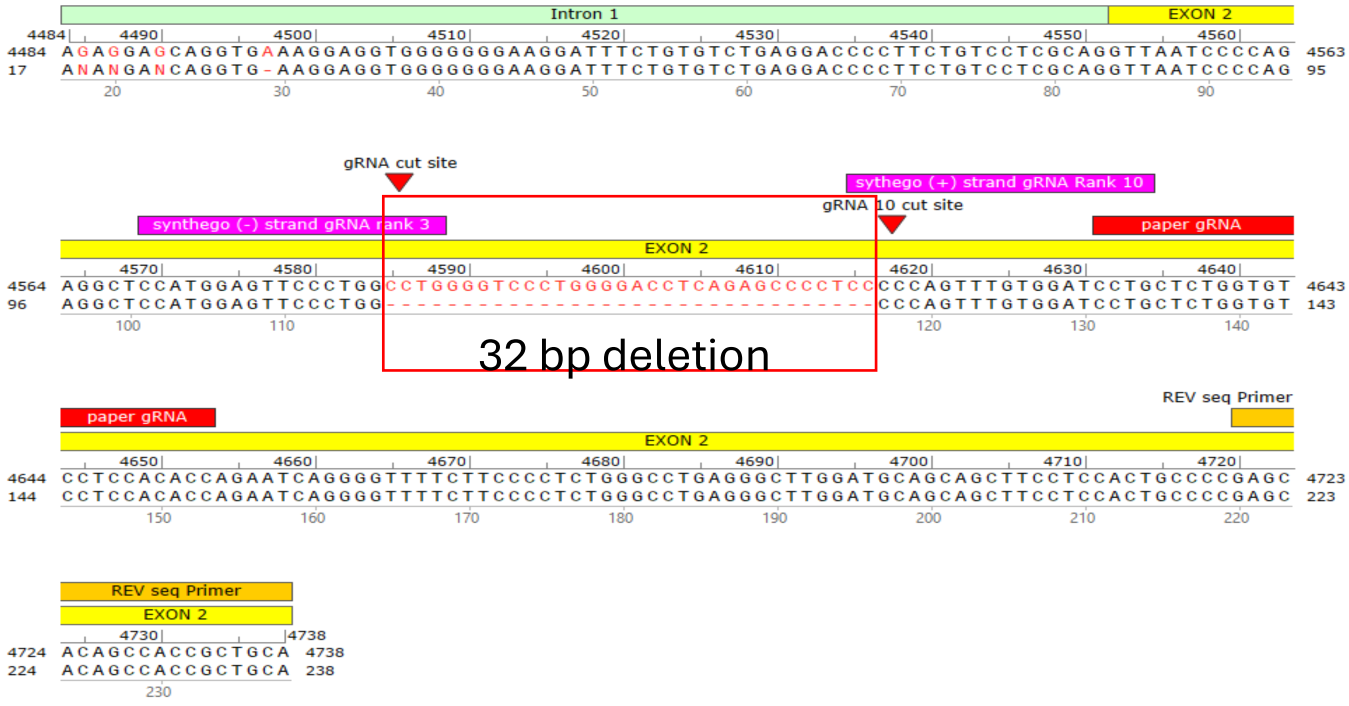

bioRxiv preprint doi: https://doi.org/10.1101/2024.10.11.617742; this version posted October 11, 2024. The copyright holder for this preprint (which was not certified by peer review) is the author/funder, who has granted bioRxiv a license to display the preprint in perpetuity. It is made available under aCC-BY 4.0 International license.

S1 Fig: Confirmation of 32bp deletion in the ORF of *GATA1*. (A) Agarose gel showing a shift in the *GATA1* gene following CRISPR-Cas9 mediated deletion of a 32bp fragment in exon 2 of the coding sequence for the gene. (B) Sanger sequencing results confirming loss of the 32bp fragment in exon 2 of the GATA1-KO mutant.

bioRxiv preprint doi: https://doi.org/10.1101/2024.10.11.617742; this version posted October 11, 2024. The copyright holder for this preprint (which was not certified by peer review) is the author/funder, who has granted bioRxiv a license to display the preprint in perpetuity. It is made available under aCC-BY 4.0 International license.

S2 Figure

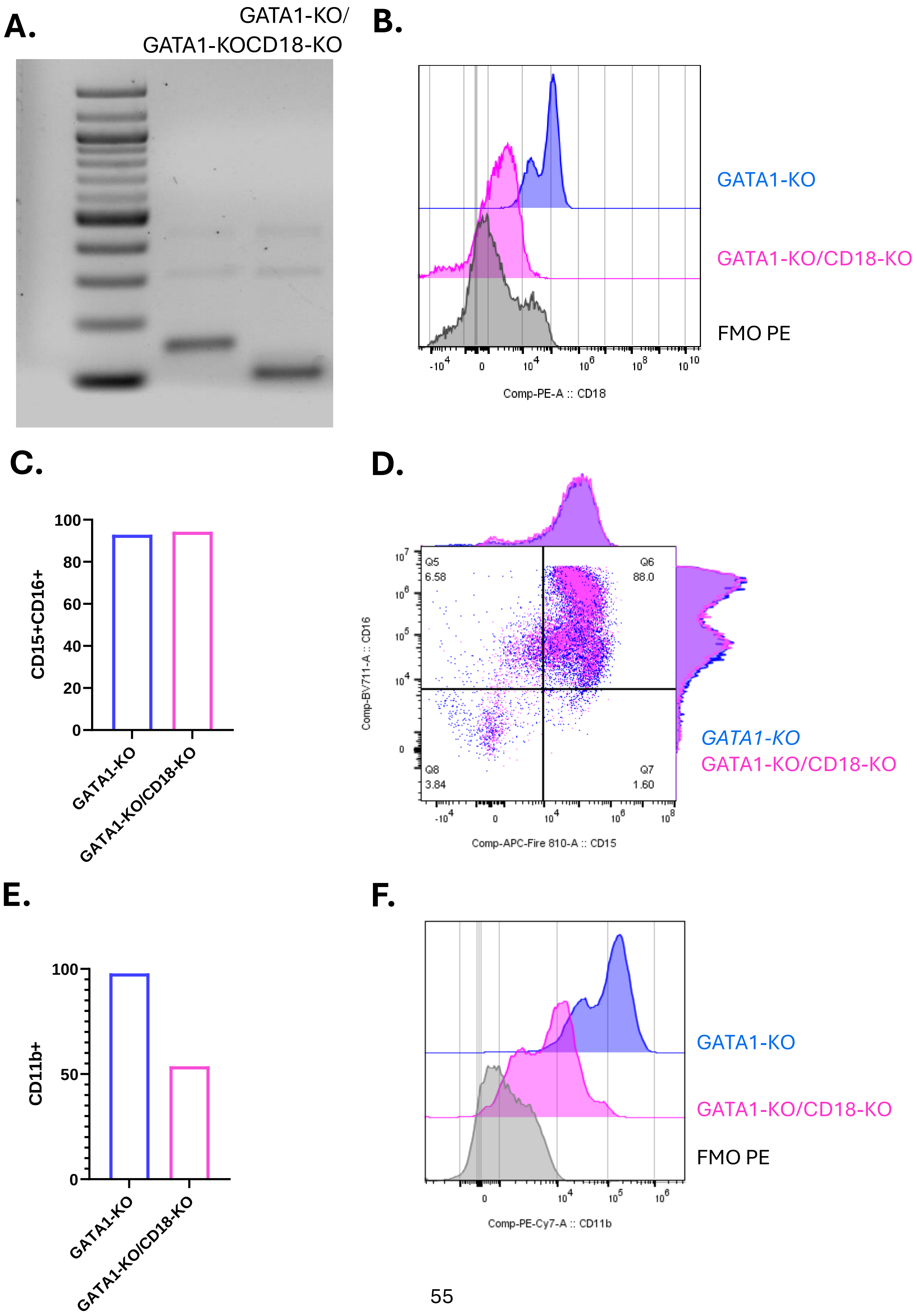

bioRxiv preprint doi: https://doi.org/10.1101/2024.10.11.617742; this version posted October 11, 2024. The copyright holder for this preprint (which was not certified by peer review) is the author/funder, who has granted bioRxiv a license to display the preprint in perpetuity. It is made available under aCC-BY 4.0 International license.

S2 Fig: Loss of CD18 reduces surface expression of CD11b. (A) Agarose gel showing a shift in the *ITGB2* gene following CRISPR-Cas9 mediated deletion of a 52bp fragment in exon 4 of the coding sequence of the gene. (B) Representative histograms of surface marker staining for CD18 in GATA1-KO and GATA1-KO/CD18-KO iNeutrophils. Fluorescence minus one (FMO) PE is indicative of a negative control for CD18 staining where the anti-CD18-PE antibody was not added to the cells. (C) Quantification of the number of CD15+CD16+ cells within the live population of iNeutrophils. (D) Representative scatter plots of CD15 and CD16 expression intensities for GATA1-KO (blue) and GATA1-KO/CD18-KO (pink) iNeutrophils. (E) Quantification of CD11b+ cells within the live population of iNeutrophils. (F) Representative histograms of CD11b expression intensities.

bioRxiv preprint doi: https://doi.org/10.1101/2024.10.11.617742; this version posted October 11, 2024. The copyright holder for this preprint (which was not certified by peer review) is the author/funder, who has granted bioRxiv a license to display the preprint in perpetuity. It is made available under aCC-BY 4.0 International license.

S1 Movie: GATA1-KO iNeutrophils aggregate around and kill *A. fumigatus in vitro*. Representative movie of GATA1-KO iNeutrophils interacting with and killing *A.fumigatus* germlings (red) during coincubation. Images were taken every 3 minutes over a 12-hour incubation period. Scale bar is 50μm. Movie is displayed as 7 frames/second.

S2 Movie: iNeutrophils migrate efficiently in response to the chemoattractant LTB4. Representative fluorescent movies of WT (left) and GATA1-KO (center) and primary human (right) (i)Neutrophils migrating towards an LTB4 gradient at the bottom of the image. iNeutrophils are stained with calcein and tracked using the fluorescent images. Cell tracks of quantified cells are overlaid and appear yellow. Scale bar is 100μm. Images were taken every 30 seconds for 45 minutes. Movie is displayed as 7 frames/second.

S2 Movie: Loss of CD18 impairs GATA1-KO iNeutrophil migration efficiently to the LTB4. Representative fluorescent movies of GATA1-KO (left) and GATA1-KO/CD18-KO (right) iNeutrophils migrating towards an LTB4 gradient at the bottom of the image. iNeutrophils are stained with calcein and tracked using the fluorescent images. Cell tracks of quantified cells are overlaid and appear yellow. Note the reduced track length of GATA1-KO/CD18-KO cells compared to their parental control. Scale bar is 100μm. Images were taken every 30 seconds for 45 minutes. Movie is displayed as 7 frames/second.

bioRxiv preprint doi: https://doi.org/10.1101/2024.10.11.617742; this version posted October 11, 2024. The copyright holder for this preprint (which was not certified by peer review) is the author/funder, who has granted bioRxiv a license to display the preprint in perpetuity. It is made available under aCC-BY 4.0 International license.

Supplementary Table 1: Antibodies used in this study.

Target	Fluor	Clone	Catalog #	Vendor
CD11b	PeCy7	ICRF44	301321	Biolegend
CD15	APC-Fire 810	W6D3	323058	Biolegend
CD16	BV711	3G8	563127	BD Biosciences
BLT1R	BUV805	14F11	749044	BD Biosciences
Clec7a (Dectin-1)	APC	15E2	355405	Biolegend
TLR2	FITC	W15145C	392307	Biolegend
TLR4	PE	HTA125	312805	Biolegend
CD18	PE	CBRLFA-1/2	366304	Biolegend
CD32	FITC	FUN-2	303204	Biolegend
Zombie NIR	746	NA	423105	FischerScientific
Human TruStain FcX Fc Receptor Blocking Solution	NA	NA	422302	Biolegend
Ultracomp eBeads	NA	NA	501129040	ThermoFisher
